# Fibroblast-to-myofibroblast transition in bronchial asthma

**DOI:** 10.1007/s00018-018-2899-4

**Published:** 2018-08-12

**Authors:** Marta Michalik, Katarzyna Wójcik-Pszczoła, Milena Paw, Dawid Wnuk, Paulina Koczurkiewicz, Marek Sanak, Elżbieta Pękala, Zbigniew Madeja

**Affiliations:** 1 Department of Cell Biology, Faculty of Biochemistry, Biophysics and Biotechnology, Jagiellonian University, Gronostajowa 7, 30-387 Kraków, Poland; 2 Department of Pharmaceutical Biochemistry, Faculty of Pharmacy, Jagiellonian University Medical College, Medyczna 9, 30-688 Kraków, Poland; 3 Division of Molecular Biology and Clinical Genetics, Department of Medicine, Jagiellonian University Medical College, Skawińska 8, 31-066 Kraków, Poland

**Keywords:** Fibrosis, Lungs, TGF-β-signalling, Pro-fibrotic agents, Mechanical forces

## Abstract

Bronchial asthma is a chronic inflammatory disease in which bronchial wall remodelling plays a significant role. This phenomenon is related to enhanced proliferation of airway smooth muscle cells, elevated extracellular matrix protein secretion and an increased number of myofibroblasts. Phenotypic fibroblast-to-myofibroblast transition represents one of the primary mechanisms by which myofibroblasts arise in fibrotic lung tissue. Fibroblast-to-myofibroblast transition requires a combination of several types of factors, the most important of which are divided into humoural and mechanical factors, as well as certain extracellular matrix proteins. Despite intensive research on the nature of this process, its underlying mechanisms during bronchial airway wall remodelling in asthma are not yet fully clarified. This review focuses on what is known about the nature of fibroblast-to-myofibroblast transition in asthma. We aim to consider possible mechanisms and conditions that may play an important role in fibroblast-to-myofibroblast transition but have not yet been discussed in this context. Recent studies have shown that some inherent and previously undescribed features of fibroblasts can also play a significant role in fibroblast-to-myofibroblast transition. Differences observed between asthmatic and non-asthmatic bronchial fibroblasts (e.g., response to transforming growth factor β, cell shape, elasticity, and protein expression profile) may have a crucial influence on this phenomenon. An accurate understanding and recognition of all factors affecting fibroblast-to-myofibroblast transition might provide an opportunity to discover efficient methods of counteracting this phenomenon.

## Introduction

Bronchial asthma is one of the most common chronic diseases in the world. It affects over 10% of the human population, and its prevalence is still rising. Bronchial asthma is a clinically heterogeneous, chronic inflammatory disorder of the airways characterized by their hyperresponsiveness to environmental stimuli and by airflow limitation. The regulatory mechanisms and consequences of inflammation in asthma form a complicated network of reciprocal influences, including a sequence of events through which structural and infiltrating cells and their signalling molecules are involved in the irreversible rebuilding of the bronchial wall (called remodelling) [1, 2]. Airway remodelling is defined as a sequence of chronic structural changes that lead to thickening of the airway wall, epithelial damage, subepithelial fibrosis, increased deposition of extracellular matrix (ECM), smooth muscle hypertrophy, and increased vascularity [3–6]. Severe asthma, as defined by the clinical presentation, is most strongly associated with remodelling. However, inflammatory cell subtypes in asthma are also relevant to the thickening of the bronchial wall. Eosinophilic inflammation of the airways was found to correlate with the loss of lung function due to a decline in the FEV1/FVC ratio [7]. However, in a recent study, transgenic expression of interleukin-8 in bronchial epithelium mimicked a severe asthma phenotype in mice and induced the neutrophilic phenotype and progressive remodelling of the airways [8].

Despite extensive research, several important questions concerning the pathogenesis of asthma remain unanswered. It is not clear whether airway remodelling is a normal response to chronic inflammation or, alternatively, whether the remodelling process itself may be a primary event in asthma development independent of inflammation [6]. Some evidence shows that airway inflammation is not the only cause of remodelling. First, changes in the bronchial wall can occur in early childhood, not necessarily subsequent to, but rather before inflammation [9–11]. Second, drugs specifically targeting inflammatory pathways that are commonly used in asthma treatment have had limited or no success in suppressing bronchial wall remodelling [12–14]. Moreover, many recent population and epidemiological studies have indicated that hereditary factors are very important in the development of asthma and in bronchial wall remodelling [15–20]. It is well documented that the lungs of patients with asthma are characterized by airway narrowing and increased thickness of the airway wall (thickening of muscle bundles and subepithelial fibrosis), which correlate with the severity of bronchial asthma [21, 22]. Subepithelial fibrosis occurs in the airway mucosa, which contains mainly fibroblasts, myofibroblasts, inflammatory cells, vessels and ECM proteins [3, 5]. The thickening of muscle bundles results from hyperplasia and hypertrophy of airway smooth muscle cells (ASMC) and their distinct hyper-reactive (‘primed’) phenotype, which are characterized by increased release of pro-inflammatory and immunomodulatory factors [6]. The key role of ASMC in remodelling has been intensively investigated and fairly well clarified [6, 23–29]. The thickening of the asthmatic (AS) subepithelial layer is due to exaggerated deposition of ECM proteins (primarily collagen I, III, and V and non-collagenous proteins, including elastin, tenascin, fibronectin and laminin), which are predominantly produced by activated ASMC, fibroblasts and myofibroblasts [30–34]. To complete the picture of events occurring in AS bronchial walls, fibroblasts, myofibroblasts and their interactions should also be considered. These remarkable cells appear to be crucial for the changes leading to narrowing of the airway lumen. The contribution of myofibroblasts to the progression of bronchial wall remodelling in asthma is indisputable, but the role of fibroblasts in the subepithelial layer in myofibroblast transition, although frequently described, remains ambiguous. In this review, we aim to assemble the current knowledge on components and processes that may lead to myofibroblast formation, especially as a result of fibroblast-to-myofibroblast transition (FMT) in bronchial asthma.

## Myofibroblasts in the bronchial wall

Myofibroblasts are mesenchymal cells that, due to their phenotype, are often described as a cross between fibroblasts and smooth muscle cells. Myofibroblasts are able to synthesize ECM proteins (as are fibroblasts) and the myocyte-specific isoform α-smooth muscle actin (α-SMA), which is visible in cells as stress fibres. These features enable myofibroblasts to induce a contractile force. It is generally accepted that myofibroblasts (including bronchial myofibroblasts from AS individuals), in addition to their expression of α-SMA, express transgelin (SM-22-α), smooth muscle myosin, osteopontin, and calponin-1 and are interconnected via gap junctions, highlighting their similarities with smooth muscle cells. As mesenchymal cells, myofibroblasts express vimentin and fibroblast surface protein (FSP) [35–41]. The contractile apparatus of myofibroblasts is composed of α-SMA-enriched bundles of microfilaments terminated with focal adhesions (FAs) positive for integrins (α1, α3, α4, α5, αV, β1), vinculin, paxillin, talin, and tensin [42–45]. It was shown that compared to fibroblasts, human bronchial myofibroblasts have a larger mean surface area and reduced extension of cell shape (extension is a measure of how much the shape differs from a circle, taking a value of zero if the shape is circular and increasing without limit when the shape becomes less circular) [46]. Human bronchial fibroblasts (HBFs) are generally smaller and less elongated than mature myofibroblasts [46–48].

Bronchial myofibroblasts are not only contractile but also metabolically active. AS myofibroblasts display increased expression and secretion of ECM components, such as collagens I, III, and V, fibronectin [49, 50], tenascin [51] and proteoglycans (lumican, versican biglycan and decorin) [50, 52, 53]. Enhanced collagen production by fibroblasts and myofibroblasts leads to greater thickness of the lamina reticularis in bronchi of AS patients (between 4 and 12 μm, in comparison with 2–6 μm in non-asthmatic (NA) subjects) [49, 54–56]. Chu et al. suggest that although increased collagen deposition in the subepithelial basement membrane is a characteristic of asthma, it may not explain the differences in severity of asthma [57]. It is known that myofibroblasts are also a source of matrix metalloproteinases (MMPs) and their inhibitors (tissue inhibitors of metalloproteinase, TIMP) [33, 58, 59]. In bronchoalveolar lavage fluid (BALF), sputum, and airway biopsies from AS subjects, increased MMP-9 and TIMP-1 expressions were demonstrated [60–62]. However, compared to control subjects, AS subjects have a significantly lower MMP-9 to TIMP-1 ratio, which correlates with the degree of airway obstruction. Weitoft and co-workers demonstrated that in both controlled and uncontrolled asthma, the MMP-9/TIMP-3 ratio is decreased [50]. Many reports have also shown that myofibroblasts are an abundant source of inflammatory mediators, cytokines, chemokines, and growth factors, such as granulocyte–macrophage colony-stimulating factor (GM-CSF), interleukins (IL-1, IL-6, IL-8), stem cell factor (SCF), transforming growth factor type β (TGF-β), and vascular endothelial growth factor (VEGF) [63–66]. Thus, myofibroblast-derived factors may act not only on themselves but also on other airway and immune cells, such as smooth muscle cells, by promoting cell migration, hyperplasia and hypertrophy [67, 68].

Several sources of myofibroblasts have been identified thus far. Myofibroblasts may arise as a result of both epithelial-to-mesenchymal transition (EMT) [69–72] and endothelial-to-mesenchymal transition (EndoMT) [73]. Fibrocytes and mesenchymal stem cells circulating in the blood and originating from bone marrow may also be a source of myofibroblasts [74–81]. Fibrocytes are an important source of myofibroblasts in chronic severe asthma [82]. Myofibroblasts can also be derived from differentiated pericytes [83, 84] or smooth muscle cells [40]. However, the most common source of myofibroblasts is the population of fibroblasts residing in the connective tissue of bronchi, as under the influence of various stimuli, fibroblasts can change their phenotype to that of myofibroblasts.

## FMT

FMT is a phenomenon that occurs in the human body under both physiological and pathological circumstances. An increase in myofibroblast formation in the connective tissue as well as disturbances in apoptosis is related to impaired wound healing and chronic inflammation. Thus, abnormal myofibroblast formation is often described in the pathogenesis of fibrotic diseases. Enhanced formation of myofibroblasts has also been reported in subepithelial remodelling in asthma [85, 86].

The primary mechanism of FMT has been discovered and described in wound healing [87]. Numerous in vitro studies have suggested that the FMT process requires two stages. In the initial phase, fibroblasts develop a transitional phenotype known as proto-myofibroblasts, which are then converted into fully differentiated (mature) myofibroblasts [42, 88]. Fibroblast-to-proto-myofibroblast transition is facilitated by mechanical tension within the wound and is accompanied by ED-A fibronectin [89] and platelet-derived growth factor (PDGF) secretion. PDGF is able to induce the formation of stress fibres and increase the motility of cells [90]. The formed proto-myofibroblasts express both β- and γ-actin isoforms (incorporated into stress fibres) and N-cadherin, which exert less adhesion force than OB-cadherin but facilitate the increased motility of proto-myofibroblasts [91]. Distinguishing between fibroblasts and proto-myofibroblasts is very difficult in vitro because most of the fibroblasts in culture exhibit a proto-myofibroblast phenotype [88]. A prolonged state of high stress and the presence of FMT-stimulating cytokines, growth factors and ECM proteins cause proto-myofibroblasts to initiate synthesis of α-SMA and gradually form α-SMA-containing stress fibres. Fully differentiated myofibroblasts express OB-cadherin, possess mature FAs (containing de novo expression of focal adhesion kinase (FAK) and tensin), and exhibit less motility, a reduced proliferation rate and enhanced contractility [42, 64, 91].

In asthma and other fibrotic lung disorders, FMT proceeds in a similar manner, but its effect on the bronchi microenvironment seems to be different. Typically, myofibroblasts enter the apoptosis pathway after fulfilling their function. Some in vitro studies have suggested that normal lung myofibroblasts can differentiate back into fibroblasts [92, 93]. In asthma, myofibroblasts seem to remain within the tissue and actively participate in bronchial wall remodelling by inducing a contractile force on the surrounding cells and ECM as well as by secreting growth factors and ECM components [94].

## Stimuli affecting FMT in asthma

Previous studies on the nature of FMT have led to the identification of various factors involved in the induction of this phenomenon in asthma. The humoural agents primarily include growth factors, cytokines and chemokines. The second group of FMT-triggering agents are mechanical factors, among which intercellular interactions and the interaction of cells with different substrates and ECM proteins should be distinguished. Due to the complicated pathogenesis of asthma, many FMT stimuli may interact with one another, thereby leading to further induction of FMT. The most important and best-described FMT-triggering factors in asthma are summarized in Table 1.

**Table 1:** Overview of factors affecting FMT in asthma

Humoural factors			
Growth factors	Interleukins	Chemokines	Others
TGF-β (β1, -β2) [95, 97, 98, 118−128] CTGF [126–128, 135] PDGF [90, 136, 137] NGF [139–141]	IL-4 [146–151, 154, 155, 157] IL-13 [146–154, 156–159] IL-5 [150] IL-11 [160, 161] IL-17 [162–166] IL-25 [167–170] IL-33 [171, 172] Oncostatin M [173] TNF-α [118]	Osteopontin [184] Eotaxin-1 [183] Eotaxin-2 [182, 185] Eotaxin-3 [182, 185] Periostin [154, 187, 198]	Bradykinin [204] Cysteinyl leukotrienes (LTD4) [202, 203] Fizz1 [199–201] Endothelin-1 [205, 206]

Mechanical factors	ECM proteins
Mechanical forces/stress [91, 208–211, 218–222] Substrate stiffness [212, 217] Cell–cell adhesions [44, 223] Epithelial cells with an asthmatic phenotype [34, 224]	Fibronectin domain (ED-A) [226] Tenascin [229]

### Humoural factors

According to the current literature, the role of growth factors in triggering FMT is unquestionable and fundamental. Among all the identified pro-fibrotic factors, the best known is TGF-β. Three homologous isoforms of TGF-β have been identified (TGF-β_1_, TGF-β_2_ and TGF-β_3_). TGF-β is secreted into the extracellular space by both bronchial structural cells (epithelial cells, fibroblasts, endothelial cells, vascular cells and ASMC) and inflammatory cells infiltrating the bronchial wall (eosinophils, macrophages) [95–98]. Literature data indicate that all TGF-β isoforms are secreted in the AS lung, but among them, the β_1_ and β_2_ isoforms seem to be the most significant [99–103]. Increased levels of TGF-β in the bronchi [95, 104] and BALF of AS subjects have been described [105, 106]. The existence of a relationship between the amount of TGF-β present in the respiratory tract and the severity of asthma has also been suggested [106, 107]. Nevertheless, several studies investigating the expression of TGF-β_1_ in asthma have shown conflicting results. It has been demonstrated in human bronchial biopsy specimens that there are no differences in the immunohistochemical staining of TGF-β_1_ between AS and control subjects [56, 108, 109]. However, TGF-β has been confirmed to play an important role in most cellular biological processes leading to airway remodelling in asthma. TGF-β affects different cell types and exhibits pleiotropic and immunomodulatory functions [95, 106, 110–115]. Depending on the chemical and mechanical conditions, TGF-β may have pro- or anti-apoptotic effects on epithelial cells [116] and can induce EMT in airway epithelial cells from AS subjects [117]. It is well documented that TGF-β is able to trigger FMT in AS subjects both in vitro [95, 97, 98, 118, 119] and in vivo [120–122]. It was also shown that in AS subjects, TGF-β can indirectly contribute to fibrosis by triggering the production of other fibrosis mediators, such as interleukin-6 (IL-6) [123]. TGF-β can also induce or enhance the secretion of fibroblast growth factor-2 (FGF-2), connective tissue growth factor (CTGF) and VEGF from fibroblasts, myofibroblasts or airway smooth muscle [124–128].

CTGF (also known as CCN2) is another important growth factor that participates in fibrotic processes in bronchial asthma. CTGF is also involved in the course of chronic inflammatory diseases [129] and is overexpressed in the lung tissue and plasma of AS subjects [130]. This growth factor in bronchi is mainly produced by fibroblasts, epithelial cells, endothelial cells and ASMC. The role of CTGF in bronchial wall remodelling, similar to that of FMT, mainly involves mediating many of the effects of TGF-β [126–128, 131]. For example, it was demonstrated that TGF-β-induced CTGF release can enhance fibronectin, collagen I and VEGF production by ASMC [132–134] as well as induce FMT [126–128, 135].

There is no doubt that in addition to the well-coordinated activities of TGF-β and CTGF, other growth factors directly or indirectly participate in FMT induction in asthma. PDGF, for example, increases the migration and phenotypical shifts of lung fibroblasts from AS patients [90] and has been shown to induce procollagen I expression in lung fibroblasts derived from patients with severe asthma [136] and increase the lung fibroblast proliferation rate in AS subjects [137]. In turn, nerve growth factor (NGF), the level of which is elevated in AS airways, [138] is able to induce fibroblast activation, fibronectin-induced fibroblast migration, and α-SMA and matrix contraction in pulmonary fibroblasts [139–141]. It also seems that basic fibroblast growth factor (bFGF), PDGF and insulin growth factor 1 (IGF-1) can positively regulate lung fibroblast proliferation in asthma [118, 142].

Other substances that are of great importance in triggering FMT are pro-inflammatory cytokines and chemokines (Table 1). Inflammation clearly plays a key role in asthma pathogenesis [1, 143–145]. An elevated influx of immune cells is associated with increased vascular permeability, and a release of cytokines and chemokines has been observed in AS airways during disease exacerbation. Presumably, interleukins, including IL-4 and IL-13, are strongly associated with inflammatory reactions in asthma. The participation of interleukins in FMT is also quite well understood. IL-4 and IL-13 may directly act on lung fibroblasts and induce myofibroblastic transition through the downregulation of cyclooxygenase (COX) gene expression and reduction of prostaglandin E_2_ production [146]. Moreover, IL-4 and IL-13 can induce FMT through the c-Jun NH2-terminal kinase-dependent pathway [147]. In addition, both interleukins are important players in the induction of the myofibroblast phenotype [148–159]. How other interleukins affect the induction of FMT in asthma has not been explained, but several in vitro and in vivo studies have suggested that many interleukins may increase the potential of FMT in a TGF-β-dependent or TGF-β-independent manner. For example, cytokines that can indirectly and directly induce FMT are IL-5 [150], IL-11 [160, 161], IL-17 [162], IL-17A [163–166], IL-25 [167–170], IL-33 [171, 172], tumour necrosis factor type α (TNF-α) [118], interleukin-6 (IL-6) superfamily members, and oncostatin M (OSM) [173]. All of the above cytokines have also been reported to be overexpressed in asthma [150, 174–180].

In the group of chemokines, special interest within the context of FMT induction should be paid to eotaxins (eotaxin-1, eotaxin-2 and eotaxin-3) [181–184], osteopontin (OPN) [39, 185, 186], and periostin [154, 187–190]. First, OPN is upregulated in asthma and associated with bronchial remodelling in humans. In addition, increased subepithelial expression of OPN correlates with disease severity [39, 186]. In mice, OPN has been demonstrated to induce the transition of lung fibroblasts into myofibroblasts [185]. Another set of chemokines, eotaxins, can selectively modulate lung and bronchial fibroblast activity by increasing fibroblast proliferation and by regulating MMP-2 activity, collagen synthesis, and cell migration [182, 183]. Recently, there has been particular interest in periostin as a pro-fibrotic factor in asthma [154, 188–191]. This biomarker of eosinophilia and type 2 inflammation in asthma is produced mainly by epithelial cells, fibroblasts, eosinophils, and fibrocytes [103, 154, 192–196]. The participation of periostin in the promotion of fibroblast transition into myofibroblasts and the induction of fibroblast migration has been described [187, 197]. It is also possible that periostin, as a co-factor of TGF-β, promotes ECM production and FMT [154, 187, 198].

In the group of humoural factors that induce FMT, attention should also be paid to unclassified factors such as Fizz1 [199–201], cysteinyl leukotrienes [202, 203], bradykinin [204] and endothelin-1 (ET-1) [205, 206]. All of these factors can also act directly on myofibroblast formation.

### Mechanical factors

The second group of FMT-inducing factors (Table 1) includes mechanical factors. It is well known that the state of mechanical tension and changes in the tissue microenvironment are crucial for FMT efficiency. Physical alterations involved in the formation of myofibroblasts in a variety of tissues, including lung tissues, have been investigated for more than 10 years [40, 45, 88, 207]. A number of studies both in vitro (with fibroblasts in 2D culture on different surfaces or in 3D collagen gels with different stiffness) and in vivo (with animal models) have shown that mechanical stress is one of the most potent factors controlling fibroblast phenotypical shifts and cell fate [208–212]. Hinz and co-workers demonstrated that proto-myofibroblasts may arise only on substrates exhibiting an elastic modulus of at least 3000 Pa and that sometimes, even stiffer culture substrates with a Young’s modulus of 20 kPa or higher are required to permit further myofibroblast transition [212]. It was also shown that the stiffness threshold for myofibroblast differentiation in vitro during wound healing is approximately 25–50 kPa [207]. As measured by atomic force microscopy, fibrotic lung tissue is up to 30 times stiffer than normal lung tissue (the Young’s modulus ranges between 20–100 and 1–5 kPa, respectively) [213–216].

It is worth emphasizing that there are few studies on the direct impact of “mechanical forces” on the lung or bronchial FMT in asthma. The exception is the study by Shi and colleagues, which showed that TGF-β_1_-induced bronchial FMT as well as cell stiffness and contractility was enhanced by increasing substrate stiffness in culture [217]. In contrast, many reports have described the effect of mechanical forces on airway remodelling in asthma [218, 219]. Numerous studies have shown an increase in ECM protein and proteoglycan content (versican, decorin, collagen I and III), MMP-2 and MMP-9 synthesis, and IL-6 and IL-8 production in AS fibroblasts under mechanical stress [218–222].

A study of bronchial and transbronchial biopsies from AS subjects revealed that patients with uncontrolled asthma have significantly increased numbers of myofibroblasts (in central airways and alveolar parenchyma) and different compositions of ECM proteins compared to patients with controlled asthma [50]. As described by Weitoft and others, the features and resulting differences in elasticity may be partly responsible for the mechanical properties of lung tissue in subjects with uncontrolled asthma. One explanation for the increased rigidity and reduced flexibility within bronchi in asthma may be the increased deposition of ECM proteins. It is well known that a local increase in matrix stiffness after injury causes an increase in the number of myofibroblasts (or FMT), which in turn results in increased secretion of ECM proteins (mainly collagens), finally causing an increase in stiffness of the ECM [40, 212]. The outcome of this feedback loop determines fibroblast morphology and actin cytoskeleton architecture and, consequently, affects the FMT process, wherein α-SMA incorporation into stress fibres, increases in the size of FAs and increases in cell contractility are observed [212]. It has been previously shown that in contrast to fibroblasts from NA bronchi, fibroblasts from AS bronchi cultured in vitro in serum-free medium, thus ensuring a lack of cell–cell interactions, are characterized by numerous, extremely thick and prominent actin cytoskeleton and relatively large FAs. These features directly contribute to the higher stiffness of fibroblasts from AS subjects than that of fibroblasts from NA subjects [44]. Other findings have shown that the initial absence or induced loss of cell–cell adhesions in AS fibroblasts is crucial for the completion of FMT [223]. In addition, Reeves et al. reported that the interaction between fibroblasts and epithelial cells could also be essential for the changed predisposition of ECM production in fibroblasts. Increased ECM and α-SMA synthesis observed in fibroblasts co-cultured with epithelial cells from bronchi of AS subjects may be the consequence of their response to the diseased epithelial cell phenotype [34, 224]. The above-mentioned interactions play an important role in inducing FMT. Although there are few reports indicating the direct involvement of mechanical factors in asthma-related FMT, their participation in this process is undeniable.

### ECM proteins that trigger FMT in asthma

A particularly important factor in the ECM protein group for the promotion of FMT is the fibronectin splice variant ectodomain A (ED-A-FN). The level of this protein was found to be increased in asthma and other pulmonary disorders [37, 48, 225]. Lung fibroblasts from OVA-treated mice lacking ED-A-FN exhibited reduced proliferation, migration, α-SMA expression, and collagen deposition as well as impaired TGF-β_1_ and IL-13 release [226]. As it is known that ED-A-FN binds TGF-β in the ECM and that it can directly interact with cells via integrins, further explanation of its unusual role in triggering FMT in asthma is not necessary. Although there is no clear evidence of the direct effect of other ECM proteins on FMT, their possible indirect influence on myofibroblast development cannot be ignored. Thus, special attention should be paid to tenascin. This myofibroblast marker is overexpressed in asthma [51, 227, 228], and its deficiency in animal models of asthma was shown to attenuate airway inflammation and, in particular, eosinophilia, IL-5 and IL-13 levels in BALF [229]. Recently, fibulin-1, a new marker of bronchial asthma, was identified [230, 231]. This secreted glycoprotein stabilizes other ECM proteins. Considering the undeniable influence of mechanical stress on FMT, increased stability of the ECM may increase fibroblast susceptibility to FMT. Another potential indirect effect of ECM on the regulation of myofibroblast formation is related to the ability of ECM proteins to bind selective growth factors. These growth factors, which may be produced in increased quantities by AS airways, are subject to a variety of interactions with ECM proteins. The direct relationship of TGF-β binding to ECM in asthma has not yet been described, but given that the regulation of TGF-β may depend on its binding to ECM proteins [94, 232], we suggest that such an interaction could also be important in asthma-related FMT.

## Fibroblast features

**Fig. 1:**
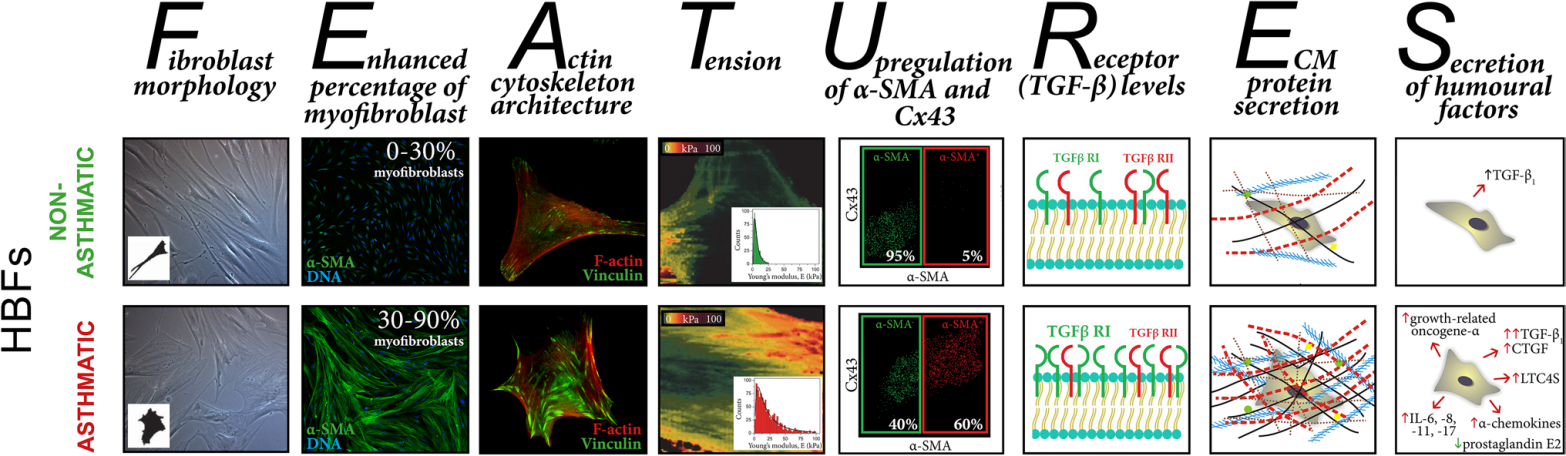
Differences in the inherent features of human bronchial fibroblasts derived from asthmatic and non-asthmatic donors that affect their potential to undergo TGF-β-induced FMT

It is generally accepted that the above-listed and described factors are crucial for FMT in asthma. However, some recently published results of in vitro studies have suggested that inherent fibroblast features can also play a significant role in this process. Michalik et al. showed that bronchial fibroblasts derived from AS patients demonstrate enhanced TGF-β_1_-induced potential to differentiate into myofibroblasts compared to their NA counterparts [46, 223], which may be attributed to the inherent features of these cells (Fig. 1).

The different properties of AS and NA HBFs that are associated with their predilection to FMT have recently been documented. Several reports have revealed significant differences in cell morphology (mainly in cell shape) between bronchial fibroblasts derived from AS and NA donors cultured under the same standard conditions [44, 46] (Fig. 1—fibroblast morphology). In addition, Kotaru et al. [233] noticed substantial differences in cell size within populations of fibroblasts isolated from proximal and distal parts of AS lungs, which correlated with their predilection to FMT. Moreover, it was shown that TGF-β_1_- or TGF-β_2_-induced FMT is accompanied by striking cell shape changes and that this phenomenon was improved in HBFs derived from AS subjects [46]. These observations correlated with an enhanced number of cells with de novo expression of α-SMA and with the incorporation α-SMA into highly contractile microfilament bundles in AS HBF populations in contrast to NA counterparts (Fig. 1—enhanced percentage of myofibroblasts) [36, 46, 204, 223, 234, 235]. Moreover, AS human lung fibroblasts (HLFs) expressed higher levels of SM22 (a protein that establishes the smooth muscle lineage of cells) than NA fibroblasts [235].

Recently, Sarna et al. used a combination of cytofluorimetric and nanomechanical analyses to demonstrate significant differences in actin cytoskeleton architecture in HBFs derived from AS patients and those derived from NA donors [44]. In contrast to NA HBFs, AS HBFs formed thick and aligned ventral stress fibres accompanied by enlarged FAs (Fig. 1—actin cytoskeleton architecture). These differences in cytoskeleton architecture between AS and NA fibroblasts correlate with the high elastic modulus and isometric tension of unstimulated (α-SMA-negative) AS HBFs (Fig. 1—tension) and their increased predilection to TGF-β-induced FMT [44].

Different behaviours of NA and AS HBFs are also observed after external stimulation. Many reports indicate that the pro-fibrotic potential of HBFs derived from AS subjects is multiplied in response to humoural and/or mechanical factors. After stimulation, AS HBFs exhibited different expression patterns of some proteins compared to NA HBFs. The most important and notable differences are amplified levels of α-SMA and connexin (Cx) 43 (protein involved in the intercellular transfer of small metabolites and ions via hexameric channels termed gap junctions) [236, 237] in response to TGF-β administration in AS HBFs compared to NA HBFs (Fig. 1—upregulation of α-SMA and Cx43) [46, 223, 234, 238]. It was shown that increased levels of Cx43 in AS HBFs correlated with their FMT potential [234]. Humoural stimulation of HBFs from AS donors induced an increased level of bradykinin B2 receptor [204], leukotriene C4 synthase and CysLT1 receptors [239], PAI-1 [235], and MRTF-A [235] but also a decreased level of prostaglandin E2 [240].

Differences in the level of TGF-β receptors were also found between AS and NA HBFs and may have an impact on FMT potential (Fig. 1—receptors TGF-β level). The results of previous studies clearly indicate that although the level of TGF-βRII in AS and NA cell populations is comparable [238], significantly increased levels of TGF-βRI in HBFs from AS subjects compared to their counterparts from healthy donors were observed [203].

Additionally, AS HBFs (through their higher tension) affect the expression of ECM components and enhance their secretion into the surrounding microenvironment (Fig. 1—ECM protein secretion). Significant differences in the expression of collagens, especially type I [158, 217, 235], proteoglycans [241], versican [221, 222], low-molecular-weight hyaluronan [242], fibronectin [235, 243], decorin [221] and tenascin C [229, 244], have been reported. Moreover, although procollagens I and III synthesis is similar in both groups of cells [137], the balance between (pro)collagen synthesis and degradation in HBFs from AS patients is unknown [245, 246]. This phenomenon is also associated with the TIMP/MMP ratio, which is unbalanced in AS HBFs [245]. These characteristics lead to the increased rearrangement and deposition of ECM components, which support the phenotypic transformation of HBFs [49, 119, 158, 217], as described earlier.

Additionally, in response to the administration of humoural factors, HBFs from AS donors secrete significantly increased levels of CTGF, IL-6, IL-8, IL-11, IL-17, α-chemokines and growth-related oncogene-α compared to their NA counterparts (Fig. 1—secretion) [106, 127, 163, 222, 247]. Similarly, the increased secretion of an active form of TGF-β_1_ is observed in both unstimulated HBFs from AS donors and HBFs under pro-inflammatory conditions [106, 158, 203]. In response to mechanical stress, HBFs from AS donors exhibit significant upregulation of IL-6, IL-8, MMP-2, MMP-9, collagen I and III expression [158, 217, 219, 222, 244]. Enhanced expression and secretion of these proteins may further auto-stimulate HBFs from AS subjects to undergo phenotypic transformation into myofibroblasts.

The differentiated nature of AS and NA HBFs (presented and summarized in Fig. 1) enabled the detection of dissimilarities in intracellular signalling pathway activity. The intensification of the output of pro-fibrotic proteins in bronchial fibroblasts from AS donors is probably dependent on the activation of different signalling pathways in comparison with their healthy counterparts.

**Fig. 2:**
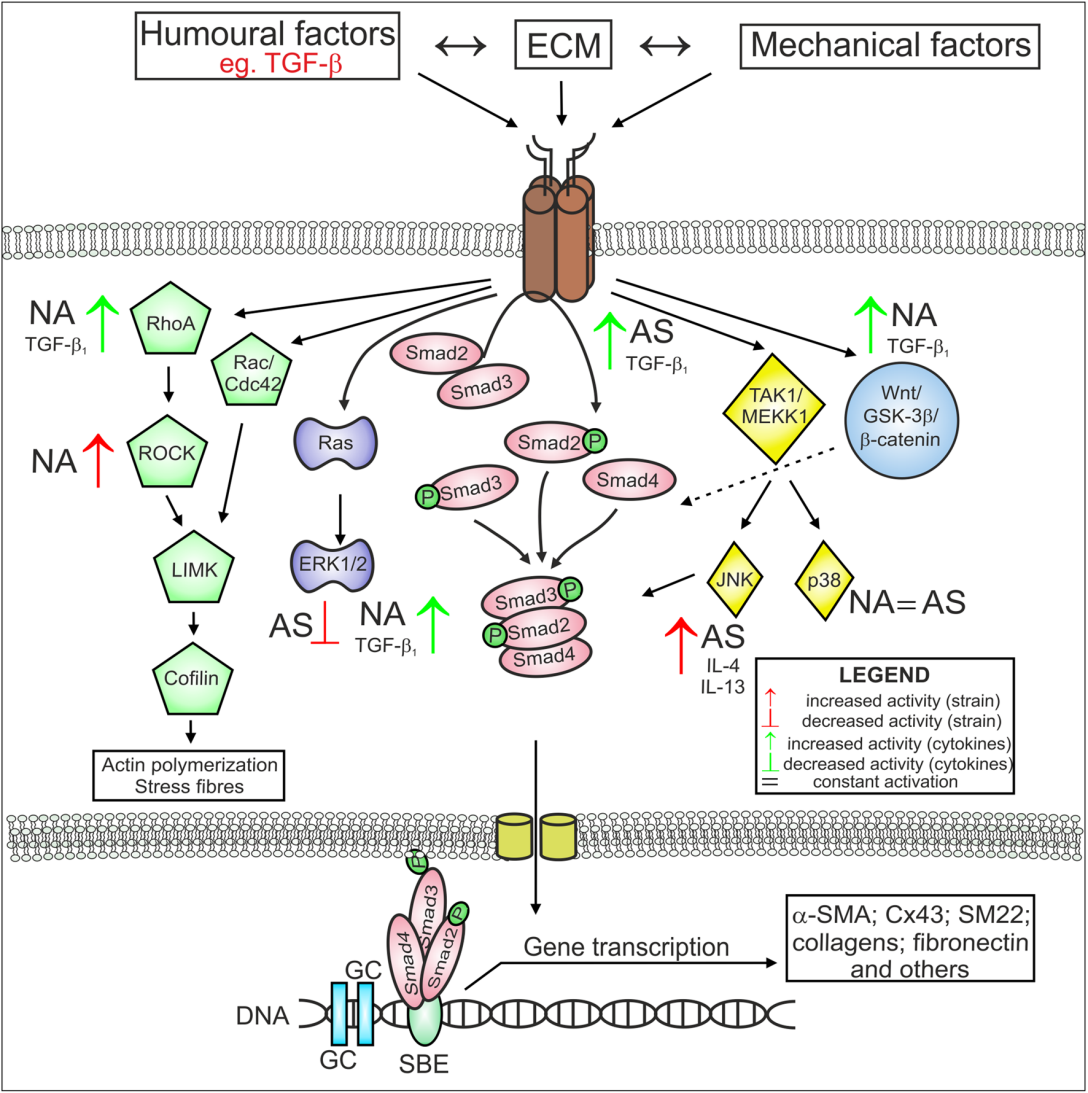
Different activation of some signalling pathways stimulated by humoural and mechanical factors in AS and NA bronchial fibroblasts. This figure is a simplified illustration of the various signalling pathways, which are, in reality, far more complex than described here (some details in the text). AS bronchial fibroblasts from asthmatic subjects, NA bronchial fibroblasts from non-asthmatic donors

Changes in ECM composition and stiffness have been shown to activate different signalling pathways (Fig. 2). Le Bellego et al. demonstrated that mechanical strain increased the secretion of pro-fibrotic and pro-inflammatory cytokines in bronchial fibroblasts obtained from AS patients, while no differences in cytokine secretion were observed in fibroblasts derived from normal volunteers [222]. Additionally, these authors revealed a mechanical strain-induced increase in ECM protein expression in only fibroblasts from AS subjects, which suggested that different signalling pathways are involved in the transduction of mechanical stimuli in AS and NA fibroblast populations [222]. In particular, mechanical stimulation of AS HBFs resulted in a concomitant increase in JNK phosphorylation and decrease in ERK1/2 phosphorylation, while p38 phosphorylation was maintained at a constant level, but during mechanical strain in NA bronchial fibroblasts, p38 phosphorylation was increased [222].

The striking difference observed in the susceptibility of fibroblasts derived from AS subjects and NA subjects to transition into myofibroblasts in response to humoural factors (mainly TGF-β_1_) indicates that these factors can facilitate TGF-β_1_-induced signal transduction in HBFs from AS patients. The differential response of AS and NA HBFs to TGF-β_1_ is mainly associated with canonical TGF-β/Smad signalling pathway activity [120, 238, 248]. Enhanced TGF-β_1_-induced Smad-dependent signalling in AS HBFs is closely linked to the increased levels of Cx43 in these cells compared to NA counterparts [234]. It has been shown that Cx43 regulates FMT through competition with Smad2 for binding sites on microtubules and acts as a type of ‘molecular switch’ [234, 249–251].

Due to the pleiotropic properties of TGF-β_1_, the induction of FMT during airway fibrosis is often associated with the activation of various non-canonical TGF-β_1_-induced signalling pathways, e.g., the mitogen-activated protein kinase (MAPK) pathway. Activation of FMT via the ERK1/2 MAPK pathway was also observed in AS fibroblasts after the administration of bradykinin [204], IL-4 and IL-13 [147] (Fig. 2). Moreover, inhibition of the p38 MAPK signalling pathway by SB203580 significantly attenuated the bradykinin-induced myofibroblastic transition of both NA and AS HBFs [204], but there are no reports concerning the effect of p38 MAPK signalling on TGF-β_1_-induced FMT in these cells. Induction of the myofibroblastic transition of NA HLFs by TGF-β is also associated with the activation of Rho-dependent signalling (Fig. 2) [235, 252]. On the other hand, it has been demonstrated that TGF-β-induced FMT in lung fibroblasts is associated with the activation of signalling via Wnt/GSK-3β/β-catenin [238, 253]. Michalik et al. found that inhibition of GSK-3β by LiCl or TWS119 attenuates TGF-β_1_-induced FMT in HBF populations derived from AS patients but not in those from healthy donors (Fig. 2). Additionally, the administration of TGF-β with inhibitors of Wnt/GSK-3β/β-catenin signalling (LiCL, TWS119) resulted in an increased level of β-catenin in NA HBFs compared to AS HBFs (Fig. 2). However, stimulation of AS HBFs by TGF-β/LiCL led to attenuation of the Smad-dependent pathway. These reports suggest that impaired intracellular trafficking of β-catenin may be involved in the differences in reactivity of AS and NA HBFs to TGF-β-induced FMT via cross-talk with Smad-dependent signalling [238]. It appears that differences in Smad- or GSK-3β/Wnt/β-catenin-dependent pathway activity in AS HBFs after TGF-β_1_ stimulation are closely associated with the cellular and molecular properties of these cells. In addition, different patterns of the above-mentioned proteins may be a response to the diverse activity of signalling pathways or may regulate their activity. However, the most likely scenario is the existence of inherent properties of cells that lead to the amplification of pro-fibrotic signals, which is supported by the intensified FMT potential of HBFs. Sources of phenotypic diversity among HBFs may be attributable to the origin of airway myofibroblast precursors.

Finally, the data mentioned above suggest that inherent lung/bronchial fibroblast features are as significant as growth factors and mechanical properties of the microenvironment surrounding the cell for the induction and effectiveness of FMT during asthma development.

**Fig. 3:**
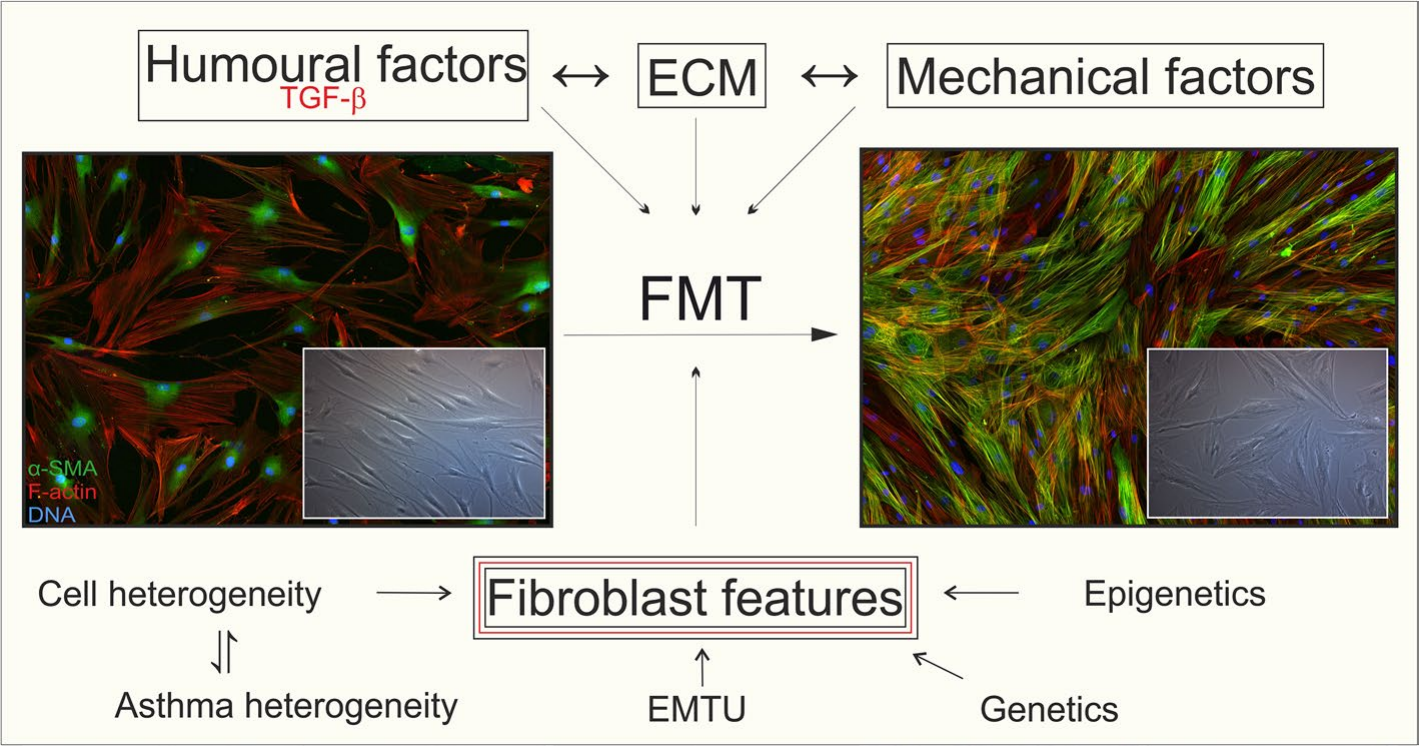
Factors affecting fibroblast-to-myofibroblast transition during airway wall remodelling in bronchial asthma. *FMT* fibroblast-to-myofibroblast transition, *TGF-β* transforming growth factor-beta *EMTU* epithelial–mesenchymal trophic unit, *ECM* extracellular matrix

Moreover, it is very important to realize that different populations of fibroblasts exist in the bronchial wall of AS subjects (Fig. 3). It was previously shown that within a single population of bronchial fibroblasts, there are cells (up to 20%) insensitive to TGF-β-induced phenotypic transition [46, 223, 238, 254]. Moreover, unstimulated AS HBF populations show an enhanced percentage of α-SMA^+^ cells compared to NA counterparts [46, 223, 238, 255, 256]. The origin of this TGF-β-insensitive population is still unexplained but may be associated with the infiltration of highly contractile myofibroblast precursors, especially CD34^+^ fibrocytes, mesenchymal stem cells, adipocytes, and pericytes, into the pro-inflammatory niche of the bronchial wall. These features may also be linked to asthma heterogeneity, showing that the multidirectional mechanisms of asthma inevitably lead to the expansion of myofibroblasts and the development of subepithelial fibrosis.

It is also important to emphasize the impact of cellular interactions on FMT potential in fibroblasts. Recent reports have indicated that AS epithelial cells favour the accumulation of myofibroblasts and stimulate ECM production in human lung fibroblast populations [34, 224]. However, little is known about the behaviour of bronchial fibroblasts co-cultured with epithelial cells, especially because different FMT potentials are observed between fibroblast populations of human bronchial and lung parenchyma (Fig. 3) [257, 258]. Finally, the effects of the differentiated features of bronchial fibroblasts can also be attributed to epigenetic or genetic factors (Fig. 3). Even though the impact of these (genetic and/or epigenetic) factors on asthma progression has been presented by others [259–262], little is known about the genetic and epigenetic factors directly affecting the differential response of bronchial fibroblasts to pro-inflammatory signals.

## Conclusion

Multiple data summarized in this article clearly indicate for the first time that the induction of FMT, a process that occurs in AS bronchial walls, requires both extrinsic (humoural, mechanical and ECM interactions) factors and inherent properties of bronchial fibroblasts (Fig. 3). Despite the yet uncertain contribution of these factors to the decline in the lung function of AS subjects, it seems practical to acknowledge that the literature supports an intervention based on topical inhibition of pathways that lead to bronchial remodelling.
